# Choice of mode of delivery in a subsequent pregnancy after OASI: a survey among Dutch gynecologists

**DOI:** 10.1007/s00192-017-3304-9

**Published:** 2017-03-22

**Authors:** Judith J. A. E. Donners, Kirsten B. Kluivers, Jan W. de Leeuw, Jeroen van Dillen, Sander M. J. van Kuijk, Mirjam Weemhoff

**Affiliations:** 1 Department of Obstetrics & Gynaecology, Maastricht University Medical Centre, P.O. Box 5800, 6202 AZ Maastricht, The Netherlands; 2 Obstetrics and Gynaecology, Radboud University Medical Centre, Nijmegen, Netherlands; 3 Obstetrics and Gynaecology, Ikazia, Rotterdam, Netherlands; 4 Clinical Epidemiology and Medical Technology Assessment, Maastricht University Medical Centre, Maastricht, Netherlands

**Keywords:** Obstetric anal sphincter injury, Subsequent pregnancy

## Abstract

**Introduction and hypothesis:**

National and international guidelines do not provide clear recommendations on the mode of delivery in a subsequent pregnancy after obstetric anal sphincter injury (OASI). The aim of this study was to investigate the opinion of gynecologists in The Netherlands on this choice and the extent to which this choice is affected by the gynecologist’s characteristics.

**Methods:**

Of 973 gynecologists sent a questionnaire seeking their opinion on the mode of delivery in 16 different case descriptions, 234 (24%) responded. Factors influencing the opinion of the respondents on the mode of delivery, the presence of anal symptoms, the degree of OASI and the characteristics of the respondents were analyzed by univariate and multivariate logistic regression analysis.

**Results:**

Recommendations on the mode of delivery in a subsequent pregnancy after OASI showed considerable variation. The recommendations depended on (previous) symptoms and the degree of OASI. For gynecologists who based their recommendations on endoanal ultrasonography outcomes (7–20% depending on the case), the degree of OASI and severity of (previous) symptoms were less important. Gynecologists basing their recommendations on endoanal ultrasonography recommended a primary cesarean section less often. Gynecologist’s characteristics (including years of experience, type of hospital and subspecialty) had a small effect on their recommendations on the mode of delivery.

**Conclusions:**

Due to lack of evidence, recommendations of gynecologists in The Netherlands on the mode of delivery in a subsequent pregnancy after OASI vary widely and depend on (previous) symptoms and the degree of OASI. Gynecologists who based their recommendations on endoanal ultrasonography outcomes recommended cesarean section less often.

## Introduction

From the literature there is no clear evidence-based recommendation that gynecologists can give to women who have sustained obstetric anal sphincter injury (OASI) on the mode of delivery in a subsequent pregnancy. This may be due to a lack of randomized controlled trials evaluating the best mode of delivery after OASI. The information available on outcomes in women with subsequent delivery after OASI is derived mainly from small, retrospective, observational studies. These studies have shown that the rate of fecal incontinence after subsequent vaginal delivery is 7% to 10% in women with a previous OASI without fecal incontinence. This rate rises to 17–40% among women with fecal incontinence after OASI [1–4]. Whether and to what extent elective cesarean section reduces the risk is unknown. Two recently published cohort studies have shown that the mode of second delivery does not significantly affect the risk of long-term anal or fecal incontinence, and concluded that vaginal delivery following OASI is safe in appropriately selected women [5, 6].

The guidelines on OASI of both the Dutch Society of Obstetrics and Gynecology (NVOG) [7] and the UK Royal College of Obstetricians and Gynecologists (RCOG) [8] recommend that the available evidence be discussed with the woman during counseling on the mode of delivery. They differ, however, with respect to the use of manometry and endoanal ultrasonography. The guidelines of the NVOG recommend that a cesarean section should not be performed just because of OASI in a previous delivery, but that this should be considered only in women with symptoms of compromised sphincter function after OASI. In contrast, the RCOG guidelines recommend that an elective cesarean section should be offered to women with symptoms of compromised sphincter function and to asymptomatic women with abnormal anorectal manometric or endoanal ultrasonographic features. The RCOG states that between 17% and 24% of women develop new or more serious complaints of anal incontinence after a second vaginal delivery [8]. The NVOG guidelines do not mention anorectal manometry or endoanal ultrasonography.

Due to the lack of high-quality evidence and clear advice in these guidelines, care-givers are left to use their best clinical judgement during counseling the woman about the best mode of delivery in subsequent deliveries. The aim of this study was to investigate the opinion of Dutch gynecologists on the best choice of mode of delivery in a subsequent pregnancy after OASI. The effect of gynecologist’s characteristics, degree of OASI, symptoms of OASI and diagnostic outcomes (ultrasonography) were assessed.

## Materials and methods

All 973 gynecologists registered in the database of the Dutch Society of Obstetrics and Gynecology were sent an online questionnaire by email between September and December 2014. All respondents included in this study worked in a general hospital and in most of them practiced obstetrics as part of their job. Physicians working in subspecialist centers, exclusively practicing general gynecology without obstetrics were not surveyed. Reminders were sent within 2 weeks to all gynecologists.

The questionnaire was developed by four gynecologists: one general gynecologist (teaching hospital), two urogynecologists (academic) and one perinatologist (academic). Prior to mass distribution, the survey was tested by five gynecologists who took on average 15 min to complete the online questionnaire. The questionnaire was divided into two parts. The first part contained questions on general characteristics of the respondent: gender, number of years of experience as a gynecologist, type of hospital, number of gynecologists in the respondent’s department and subspecialty. The second part comprised 16 case descriptions. Cases 1 to 4 described a patient without a complaint of OASI with the four different degrees of OASI as described by Sultan (grade 3A, 3B, 3C and 4 [9]. Cases 5 to 8 described a patient with transient symptoms of anal incontinence, cases 9 to 12 described a patient with persistent flatal incontinence, and cases 13 to 16 described a patient with persistent fecal incontinence. These cases were also repeated for the four different degrees of OASI. For each case description the respondent had to give a personal recommendation regarding the mode of delivery in a subsequent pregnancy: vaginal delivery, primary cesarean section or an approach depending on the outcome of endoanal ultrasonography.

The electronic questionnaire was created with the use of SurveyMonkey, a cloud-based online survey and questionnaire tool. All respondents completed the questionnaire in the SurveyMonkey database created for this purpose. All data were analyzed using SPSS version 22. The characteristics of the participating gynecologists and their recommendations on mode of delivery are presented as percentages. The effect of degree of OASI and the symptoms on the recommended mode of delivery are plotted as percentages. The associations between the characteristics of the gynecologists and the recommendation given were analyzed by univariate and multivariate logistic regression analysis. This study was approved by the medical ethics committee of the University Hospital Maastricht, Maastricht University.

## Results

Off the 973 gynecologists, 234 responded (24%). The characteristics of the respondents are shown in Table 1. Of the respondents, 59% were female and 36% had more than 15 years of experience as a gynecologist. Of all the respondents, 93% had a subspecialty, and of these, 36% were perinatologists and 31% were urogynecologists.

**Table 1:** Characteristics of the participating gynecologists

Characteristic	Number (percentage)
Gender	
Female	139 (59.4)
Years of experience	
0–5	62 (26.5)
5–10	52 (22.2)
10–15	35 (15.0)
>15	85 (36.3)
Type of hospital	
University hospital	35 (15.0)
Nonteaching hospital	116 (49.6)
Teaching hospital	83 (35.4)
Gynecologists with a subspecialty	
No	16 (6.8)
Yes	218 (93.2)
Subspecialty	
Benign gynecology	40 (18.5)
Oncology	18 (8.3)
Obstetrics	77 (35.7)
Fertility	14 (6.5)
Urogynecology	67 (31.0)

**Fig. 1:**
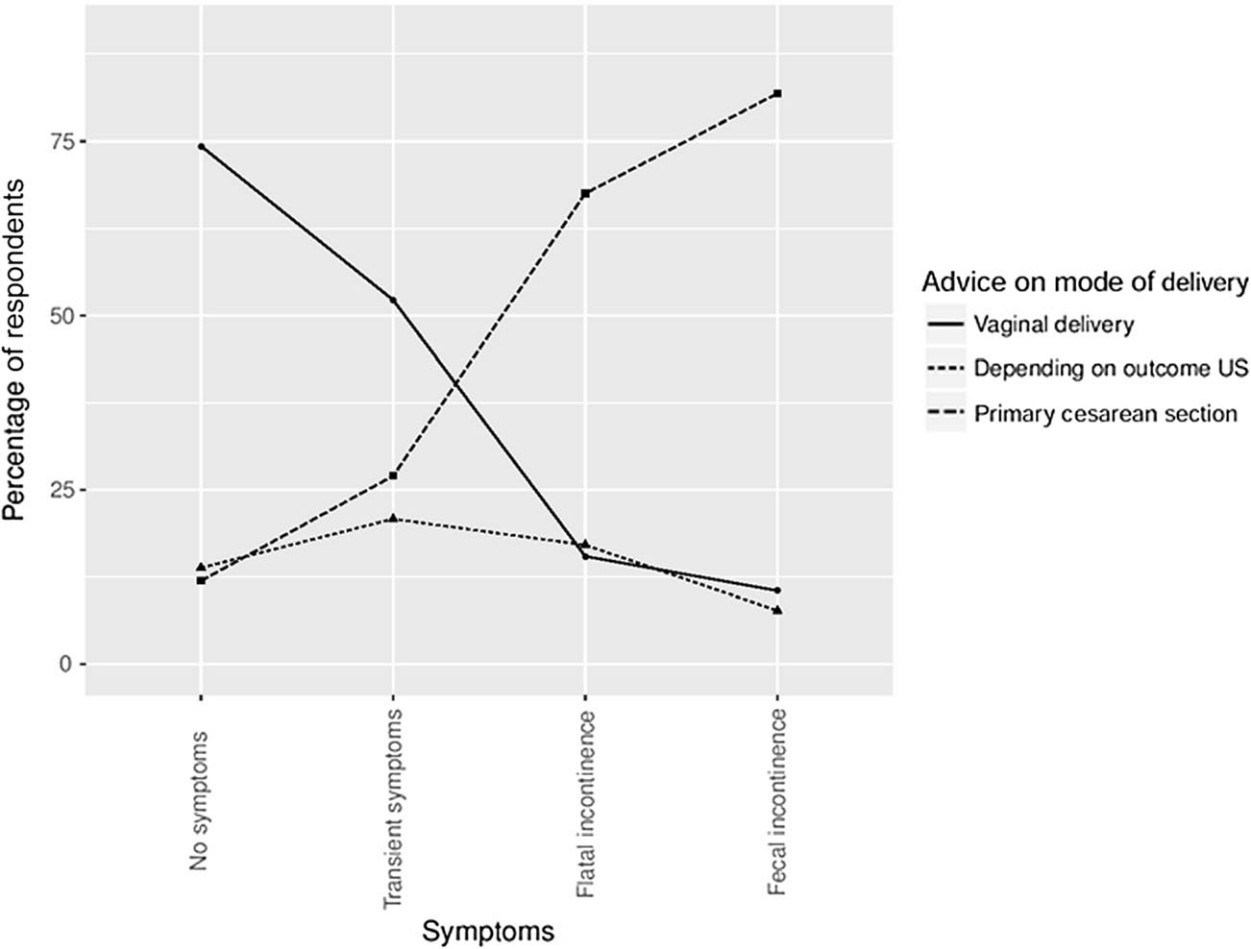
Effect of anal symptoms on the recommended mode of delivery

**Fig. 2:**
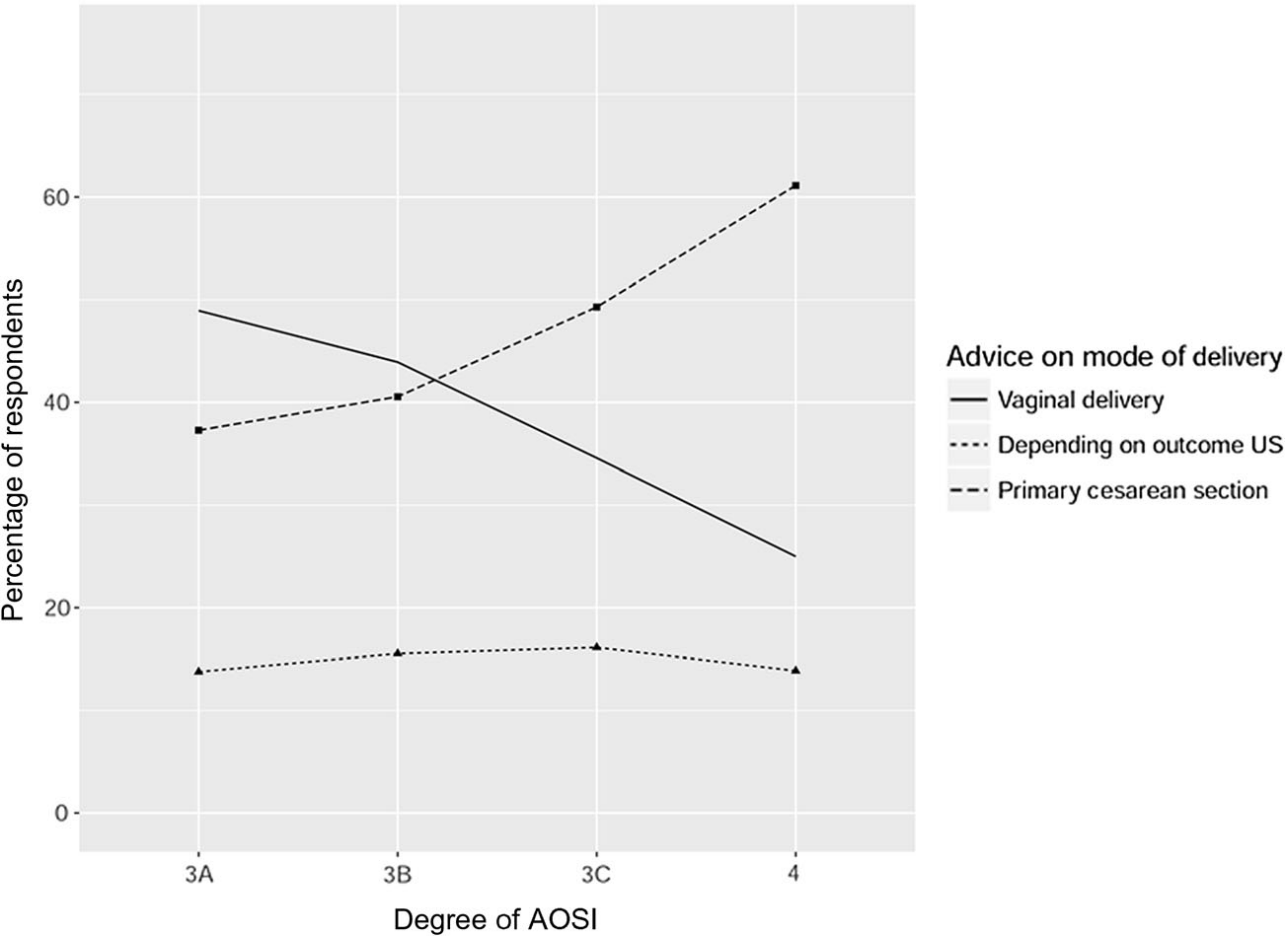
Effect of the degree of OASI on the recommended mode of delivery

The recommendations on mode of delivery in a subsequent pregnancy are shown for the 16 case descriptions in Table 2. In women without symptoms after a grade 3A perineal tear, almost 91% of gynecologists would recommend a vaginal delivery in a subsequent pregnancy. But in women with a history of a grade 4 perineal tear with persisting symptoms of fecal incontinence, almost 84% of gynecologists would recommend a cesarean section. An increasing proportion of gynecologists would recommend a cesarean section with increasing complaints of anal incontinence and increasing degree of OASI. The percentage of gynecologists who would recommend a vaginal delivery decreased from 74.3% to 10.5% with more extensive symptoms (Fig. 1). The percentage of gynecologists who would recommend a vaginal delivery decreased from 49% to 25% with increasing extent of OASI (Fig. 2). Depending on the case description, 7–20% of gynecologists would base their recommendation on endoanal ultrasonography findings. These percentages did not decrease or increase with increasing extent of OASI or with more extensive symptoms. So for gynecologists who based their recommendation on endoanal ultrasonography findings, the degree of OASI and severity of (previous) symptoms of anal incontinence had less effect on their advice.

**Table 2:** Recommendations on mode of delivery in a subsequent pregnancy

Severity of symptoms	Mode of delivery	Grade of OASI			
		3A	3B	3C	4
No symptoms	Vaginal	90.7%	86.0%	70.1%	50.5%
	Depending on ultrasonography	8.4%	12.6%	18.7%	15.4%
	Primary cesarean section	0.9%	1.4%	11.2%	34.1%
Transient symptoms	Vaginal	69.1%	60.3%	46.7%	32.7%
	Depending on ultrasonography	19.2%	22.9%	22.9%	18.2%
	Primary cesarean section	11.7%	16.8%	30.4%	49.1%
Flatal incontinence	Vaginal	23.4%	18.2%	11.7%	8.4%
	Depending on ultrasonography	19.6%	18.7%	15.9%	14.0%
	Primary cesarean section	57.0%	63.1%	72.4%	77.6%
Fecal incontinence	Vaginal	12.6%	11.2%	9.8%	8.4%
	Depending on ultrasonography	7.9%	7.9%	7.0%	7.9%
	Primary cesarean section	79.5%	80.9%	83.2%	83.7%

The characteristics of the gynecologists appeared to have an effect on their recommendation on mode of delivery. After accounting for grade of perineal tear and symptoms, the multivariate analysis showed that more years of experience, working in a nonteaching hospital and perinatologist as subspecialty were characteristics independently related to the recommendation for a vaginal delivery after an OASI (Table 3).

**Table 3:** Univariate and multivariate analysis of the effect of the characteristics of the participating gynecologists on their recommendation for vaginal delivery

Characteristic	Univariate analysis			Multivariate analysis		
	Odds ratio	95% confidence interval	*p* value	Odds ratio	95% confidence interval	*p* value
Female	0.90	0.79–1.04	0.156	0.96	0.83–1.11	0.549
Experience (>5 years)	1.34	1.15–1.58	<0.001	1.38	1.17–1.63	0.000
Nonteaching hospital	0.84	0.73–0.97	0.020	0.84	0.73–0.97	0.016
Perinatology^a^	0.92	0.84–1.00	0.042	0.91	0.83–0.99	0.023

^a^Perinatology versus urogynecology

## Discussion

The recommendation on mode of delivery in a subsequent pregnancy varied considerably among gynecologists in The Netherlands and depended on (previous) symptoms and the degree of OASI. More cesarean sections were recommended in women with higher degrees of OASI and with persistent symptoms. However, both Dutch and UK guidelines do not recommend an approach that depends on the degree of OASI. There is no evidence that higher grades of OASI are associated with a higher risk of recurrence or a higher risk of anal incontinence after a subsequent delivery. In our study Dutch gynecologists considered the degree of OASI after a previous delivery as an important factor in the decision on mode of delivery in a subsequent pregnancy. Gynecologists who based their recommendation on ultrasonography outcomes considered that the degree of perineal tear was less important than did gynecologists who did not use ultrasonography. We speculate that this is due to the fact that these gynecologists rely more on the outcome of the endoanal ultrasonography as a predictive factor than on the previously described degree of OASI. In an endoanal ultrasonography-based prospective study, Oude Lohuis and Everhardt found that the number of defecatory symptoms had a positive correlation with persistent injury [10]. Especially in women with higher degrees of OASI, a cesarean section was often recommended without ultrasonographic information available. In our study with limited information about clinical scenarios, the information from endoanal ultrasonography in women after OASI may more often lead to a trial of labor after OASI.

Transient symptoms and persistent symptoms of anal incontinence are regarded as relevant by all gynecologists when deciding on the mode of delivery in future pregnancies after OASI. Most gynecologists consider symptoms as a sign of a compromised sphincter function, leading to a recommendation in favor of cesarean section. There was a considerable difference in the recommendations in women with persistent flatal incontinence and women with transient symptoms of anal incontinence, although both conditions may be considered as (transient) compromised function of the sphincter. In women with transient symptoms of anal incontinence more gynecologists would recommend a vaginal delivery than a primary cesarean section, and in women with persistent flatal incontinence more gynecologists would recommend a primary cesarean section than a vaginal delivery. The appreciation of the clinical relevance of these conditions seem to differ and is thus not well defined.

A weakness of this study was the low response rate of 24%. We did send the questionnaire out to a large group of gynecologists. This low response rate may partly be explained by the general reminder which was send out. The gynecologists approached did not receive a personal reminder, but only a mass distributed reminder to all gynecologists. They may have felt less personally obliged to participate in the study because of the mass distributed reminder. According to age, distribution in the country and the distribution of subspecialists, our cohort of respondents appear to be a proper reflection of the entire population of Dutch gynecologists. The gender and type of hospital of the respondents corresponds roughly with the characteristics of the gynecologists in The Netherlands. In 2014, the ratio of male to female gynecologists in The Netherlands was 43% to 57% [11] compared with 41% to 59% in our study. In The Netherlands 24% of gynecologists work in a university hospital, 44% in a teaching hospital and 32% in a nonteaching general hospital [11]. In this study 15% of respondents worked in a university hospital, 50% in a teaching hospital and 35% in a nonteaching hospital. Of all participating gynecologists, 93% stated that they had a subspecialty, and of these 36% were perinatologists and 31% were urogynecologists. These percentages do not reflect the distribution of all Dutch gynecologists. There was some over-representation of urogynecologists, who were probably more interested in filling out a questionnaire on this topic. This may have led to a higher percentage of recommended primary cesarean sections.

Furthermore, in this study we did not record real-life recommendations on mode of delivery, but described different cases in a questionnaire. The responses may differ from real-life situations where patient preferences are also considered and the eventual decision is a result of shared decision making. However, in the process of shared decision making the opinion of the gynecologist plays an important role. This study explores this opinion without taking patient preferences into account. Because we did not record real-life recommendations on the use of endoanal ultrasonography, it is possible that some respondents answered the questions on endoanal ultrasonography theoretically without ever using endoanal ultrasonography data clinically. These answers may also differ from real-life situations. In The Netherlands endoanal ultrasonographic skills and equipment are not readily available in most obstetric practices. In most larger hospitals in The Netherlands it is possible to refer a patient for endoanal ultrasonography and manometry. It is possible that some participating gynecologists who have no access to endoanal ultrasonography responded more negatively, reflecting the lack of availability rather than the theoretically desired information given by endoanal ultrasonography.

In conclusion, the recommendations of Dutch gynecologists on the mode of delivery in a subsequent pregnancy varied considerably and depended not just on (transient) symptoms but also on the degree of OASI. Furthermore, the recommendation for a vaginal delivery were found to be independently associated with more years of experience, working in a nonteaching hospital and perinatology as subspecialty. Among gynecologists who based their recommendation on ultrasonography findings, the degree of OASI and severity of (previous) symptoms were less important and this group of respondents recommended primary cesarean sections less often. However, more robust evidence is required to identify the additional value of endoanal ultrasonography with regard to better outcomes.
